# Effects of a parental program for preventing underage drinking - The NGO program strong and clear

**DOI:** 10.1186/1471-2458-11-251

**Published:** 2011-04-21

**Authors:** Camilla Pettersson, Metin Özdemir, Charli Eriksson

**Affiliations:** 1 School of Health and Medical Sciences, Örebro University, S-701 82 Örebro, Sweden; 2 School of Law, Psychology and Social work, Örebro University, S-701 82 Örebro, Sweden

**Keywords:** Adolescent, underage drinking, alcohol, parental program, prevention

## Abstract

**Background:**

The present study is an evaluation of a 3-year parental program aiming to prevent underage drinking. The intervention was implemented by a non-governmental organization and targeted parents with children aged 13-16 years old and included recurrent activities during the entire period of secondary school. The program consisted of four different types of group and self-administered activities: parent meetings, family dialogues, friend meetings, and family meetings.

**Methods:**

A quasi-experimental design was used following parents and children with questionnaires during the three years of secondary school. The analytic sample consisted of 509 dyads of parents and children. Measures of parental attitudes and behaviour concerning underage drinking and adolescents' lifetime alcohol consumption and drunkenness were used. Three socio-demographic factors were included: parental education, school, and gender of the child. A Latent Growth Modelling (LGM) approach was used to examine changes in parental behaviour regarding youth drinking and in young people's drinking behaviour. To test for the pre-post test differences in parental attitudes repeated measures ANOVA were used.

**Results:**

The results showed that parents in the program maintained their restrictive attitude toward underage drinking to a higher degree than non-participating parents. Adolescents of participants were on average one year older than adolescents with non-participating parents when they made their alcohol debut. They were also less likely to have ever been drunk in school year 9.

**Conclusion:**

The results of the study suggested that Strong and Clear contributed to maintaining parents' restrictive attitude toward underage drinking during secondary school, postponing alcohol debut among the adolescents, and significantly reducing their drunkenness.

## Background

Reducing alcohol drinking among adolescents is important from a public health perspective [1,2]. An international study, including adolescents from 41 countries, showed that 44% of the participating 15-year-olds had consumed alcohol at age 13, or even earlier [3]. In Sweden, the legal age for purchasing alcoholic beverages containing over 2.25% alcohol by volume at the monopoly alcohol stores is 20, and for being served alcohol at restaurants is 18. Grocery stores are allowed to sell medium-strength beer (2.8-3.5% alcohol by volume) to adolescents over 18 years old [4]. Despite these legal restrictions, the majority of Swedish adolescents start drinking alcohol before they are 18 years old. A national survey of 15-year-old teenagers found that 58% of the Swedish girls and 65% of the Swedish boys had consumed alcohol [5]. Early alcohol debut is associated with subsequent high alcohol consumption during the whole teenage period [6]. Adolescents who start drinking at an early age are also at risk for developing alcohol dependency in adulthood [7-9]. In addition, alcohol consumption during adolescence is linked to problems such as undesired or unprotected sex, accidents, and injuries [5,10]. In Sweden, over half (53%) of the 15-year-old girls and 45% of the boys had also experienced drunkenness [5], which is highly related to injuries [11]. In sum, it is critical to postpone alcohol debut age and to prevent problem drinking, such as drunkenness, for the present and future well-being of adolescents. However, developing effective and culturally relevant interventions are major challenges for practitioners.

Even though adolescents often use alcohol outside of the home environment [e.g. [12]], previous studies have shown that family norms, attitudes, and rules about adolescents' alcohol consumption are related to drinking among young people [13-18]. The effects of positive family relationships and the role of parents in shaping children's and youths' health behaviours are well-documented [19,20]. In a review of family-based programs, all interviewed experts agreed about the importance of involving parents in substance abuse prevention programs [21]. In addition, programs targeting parents and families have been shown to be successful in preventing alcohol and drug use among adolescents [see [20-23] for reviews]. Effective interventions often aim to improve the interaction between parents and children and strengthen family bonding [15,24]. Another effective component of successful programs is encouraging parents to clarify their attitudes towards adolescents and alcohol use [15]. A Swedish study has shown that encouraging parents to adopt or maintain a zero-tolerance attitude towards youths' alcohol consumption could reduce underage drinking [25]. In conclusion, involving parents in youth alcohol use prevention programs could yield positive outcomes. Programs may focus on improving family relationships and strengthening parents' restrictive attitudes towards youth drinking.

The Swedish government has acknowledged the need to improve the existing, and develop new parental support programs. Its official stance is that the support should be based on the needs of parents and children and that participation should be voluntary [26]. Consequently, there is a growing need for research on preventive interventions. Unfortunately, intervention research is the most undeveloped domain in public health research in Sweden [27,28]. Past research has primarily focused on well-controlled efficacy studies of public health programs and little attention has been paid to studying programs under typical, rather than optimal conditions. Indeed, Green and Glasgow [29] assert that "*If we want more evidence-based practice, we need more practice-based evidence" *(p. 126).

The present study is an evaluation of "Strong and Clear" (Stark och klar), a universal parent-focused drinking prevention program aiming to maintain parents' restrictive attitudes concerning youth drinking, and in turn, to prevent alcohol drinking among adolescents. The program originates from Norway and was developed at the behest of the International Organisation of Good Templars (IOGT) in Norway [30]. The only outcome evaluation of the program, to date, used a post-test-only design with non-equivalent comparison groups, and reported that adolescents of the participating Norwegian parents had a delayed alcohol debut, drank less alcohol, and had been drunk less often than the adolescents of non-participating parents [31]. The Swedish temperance organization IOGT-NTO, which is a part of the International Organization of Good Templars, adapted the program to the Swedish context. IOGT-NTO received funds from the Swedish National Board of Health and Welfare (NBHW) to implement the program. The organization had the full responsibility for implementing Strong and Clear. An independent research team at Örebro University funded by NBHW to evaluate the program.

The purpose of this study is to examine the effects of the program on alcohol drinking among adolescents. The effects of the program on both parents and youth were measured at 15 and 27 months after the initiation of the program. The research questions were: (i) is the program effective in changing parental behaviour (allowing kids to drink at home) and attitudes towards underage drinking? (ii) is the program effective in postponing adolescent alcohol drinking? (iii) is the program effective in reducing problem drinking behaviour (i.e., drunkenness), (iv) does the program effect on youth drinking and drunkenness differ by gender of the child and parental education? and (v) is there any relation between changes in parents' attitude towards youth drinking and adolescents' drinking behaviour?

### A description of Strong and Clear

Strong and Clear is a multi-component universal program including thirteen activities during the three years of secondary school (adolescents aged 13-16). Parents could sign up for the program during the entire program period. There are four different types of group and self-administered activities, some of which involve the adolescents: *parent meetings, family dialogues, friend meetings*, and *family meetings *(see Table 1). The curriculum of the program targeted engagement across the four activities every school year except the first school year, which features an additional family dialogue [32].

**Table 1:** All thirteen activities in the program Strong and Clear by secondary school years.

Grade 7	Grade 8	Grade 9
**INFORMATION MEETING**		
- about the program		

**1. Parent meeting**	**6. Parent meeting**	**10. Parent meeting**
- youth environments, setting limits and solving conflicts. - agreements between parents, e.g. that parents will not supply alcohol to the adolescents and will react and contact other parents if adolescents are at unsuitable places or are drunk.	- facts about alcohol and drugs and the local situation. - agreements between parents.	- resources and support if something happens. - agreements between parents about how they can prevent drinking-bouts.

**2. Family dialogue**	**7. Family dialogue**	**11. Family dialogue**
- important aspects of life, to grow up and mature, and peer pressure. - agreement between parent and child, e.g. regarding alcohol and tobacco use.	- how the adolescents are feeling, alcohol and drugs, parties and peer pressure. - agreement between parent and child.	- trust and openness. - agreement between parent and child.

**3. Family meeting**	**8. Family meeting**	**12. Family meeting**
- choices, courage and motives.	- positive youth culture. - agreement between parents and adolescents.	- about the future.
- agreement between parents and adolescents.		

**4. Friend meeting**	**9. Friend meeting**	**13. Friend meeting**
- to get acquainted with the child's friends.	- a weekend evening with the child and his/her friends.	- a first-rate dinner with the child and his/her friends.

**5. Family dialogue**		
- strengths and positive characteristics.		
- agreement between parent and child.		

The *parent meetings *are intended to establish alliances between parents. They were arranged by IOGT-NTO and held in the evenings at the school. During this 2-hour meeting, parents were encouraged to discuss urgent questions with each other and to make an agreement about the topics that the parents deemed important (e.g., attitudes concerning adolescents and alcohol). The *family dialogue *was a self-administered activity where parents were sent a booklet to promote conversations at home with their child about a number of issues important during the teenage years. The parents and the child were encouraged to make an agreement about the issues that they felt important in their family. The *family meetings *were also arranged by IOGT-NTO to get parents and children meet other families at the school. *Friend meetings *were an activity where parents, adolescents, and the adolescent's friends were encouraged to engage in a recreational activity together such as eating dinner or going to bowling. The purpose was to encourage parents to get to know their teen's friends and serve as adult role models [32].

In sum, Strong and Clear is a universal multi-component program with components targeting primarily parents as well as adolescents and their peers. The program includes some important components that have been used in successful programs for reducing alcohol use among youth, such as improving family relationships [e.g. [33,34]], strengthening parents' restrictive attitudes towards youths' alcohol use and making agreements between parents [e.g. [25]].

## Methods

### Population and Sample

IOGT-NTO was funded to implement Strong and Clear in six counties in Sweden. The evaluation of Strong and Clear was concentrated to one of these counties, Värmland. IOGT-NTO was well-organized at the local level, and able to carry out the program in a sufficient numbers of schools. Six schools, located in three municipalities, were included in the study. All adolescents who started in school year 7 during autumn 2004 (n = 795) and their parents were the target sample for the evaluation study. However, the number of pupils were not consistent over the three years of secondary school due to the fact that students and families moved into and out of the area. The target group in school year 8 and school year 9 were 789 and 798 adolescents, respectively. The evaluation team administered annual questionnaires to parents and adolescents during the three years of secondary school (school year 7-9). A baseline questionnaire was mailed to all parents on the schools' mailing lists before the program was introduced to the parents. The response rates among parents were 69% at baseline, 54% in school year 8, and 46% in school year 9. The response rates were higher among the adolescents; 94%, 84%, and 79% during school year 7, 8, and 9, respectively.

In total there were 814 families where either the adolescent or the parent, or both the adolescent and the parent had answered at least one questionnaire. There was no control group in the design of program implementation. Therefore, the research team identified the parents who were not participating in the program and their children as the comparison group. For this purpose, we set two inclusion criteria: 1) parents should have responded questionnaires in Grade 8 or 9 so that we could identify whether they were participating in the program, and 2) the youths of the parents who responded questionnaires in school year 8 or 9 should participate in the questionnaires at least in once from Grade 7 to 9. The parents who did not complete any of the program activities apart from participating in the information meeting in Grade 7 served as a comparison group. This procedure has left 509 parent-youth dyads. Of the 509 parent-youth dyads, 229 (45%) of the parents were identified as program participants as they completed at least one program activity in school year 8 and 9. An attrition analysis has been carried out with logistic regression analysis. The analytical sample (n = 509) was compared to those whose parents never answered the parent questionnaire, or only answered the questionnaire in school year 7 (n = 305). First, crude odds ratios (OR) were calculated for all the measures in the study: parental education, gender of the child, schools, parents' attitude towards youth drinking, parents' behaviour regarding youth drinking, adolescents' lifetime alcohol consumption, and adolescents' drunkenness. The results suggested that adolescents not included in the study were more likely to have experienced drunkenness in school year 7 than those included (OR 1.8, 95% CI 1.0-3.3). Mothers' educational level was also a significant predictor of attrition. Mothers with secondary school as their highest level of education were more likely than mothers with university education (3 years or more) to only have answered the questionnaire in school year 7 (OR 2.3, 95% CI 1.5-3.9). Mothers who answered that they had other forms of education than secondary education or university education (2.5 years or less) were also more likely to only have answered the questionnaire in school year 7 than mothers with university education (3 years or more). There were also some differences between schools. Two additional logistic regression models were fitted to further examine the impact of attrition on the study sample. In the first logistic regression model, all socio-demographic factors were included as predictors of attrition pattern. The importance of mothers' educational level remained significant controlling for the other demographic factors.

Mothers with secondary school degree (OR 2.1, 95% CI 1.1-3.8) and mothers who attended university education for a shorter duration (2.5 years or less) (OR 2.1, 95% CI 1.0-4.2) were more likely to answer only the Grade 7 questionnaire compared to mothers with university degree. In addition, the attrition rate was different across schools. In the second model, parents' attitudes towards youth drinking, parents' behaviour regarding youth drinking, adolescents' drinking and drunkenness were added to the model. None of these variables were significant predictors of attrition pattern except across school differences.

### Ethics

The parents were informed by a cover letter describing the study procedure and they were asked to give informed consent by returning the questionnaire. In the cover letter, the parents were also asked whether they would give permission for their teenage children to participate in the annual data collections. Parents could decline to allow their child to participate by returning a form postage-free. There were few parents, who declined participation for their child (5, 4, and 6% of the parents in Grades 7, 8, and 9, respectively). The youth questionnaires were filled out in the classrooms during school hours. The adolescents were allowed to decline participation even if the parents gave consent. Both parents and adolescents were told that their participation was voluntary, and they were assured confidentiality. Neither parents nor children were paid for their participation. The same procedure for data collection was used for the follow-up questionnaires in Grades 8 and 9. The research conformed to the Helsinki Declaration and ethical approval was obtained from both Örebro University research ethic committee and the Regional Ethic Review Board at Uppsala University.

### Measures

#### Alcohol consumption

Adolescents' alcohol consumption was measured with two questions: lifetime alcohol consumption and drunkenness. In all three surveys, the adolescents were asked if they had ever drunk alcohol. The response alternatives were: (1) "have never drunk alcohol"; (2) "have taken a sip from someone else's glass"; (3) "have drunk alcohol on one occasion"; and (4) "have drunk alcohol on more than one occasion". The alternatives were dichotomized, merging alternatives 1 and 2 together, as well as 3 and 4.

The adolescents were also asked if they had ever been drunk. This question was also included in all three surveys, but the numbers of response alternatives varied. In school years 7 and 8 there were only two response alternatives (yes and no). In school year 9, the response alternatives ranged from (1) "I have never drunk alcohol" to (6) "yes, every time". The response alternatives were divided into two categories to identify (1) those who had never been drunk and (2) those who had been drunk at least once. Drinking and drunkenness questions have been used in previous research on Swedish youth [e.g., [35]].

#### Parents' attitude towards youth drinking

A single item was used to measure parents' attitude towards youth drinking (*Which of the following statements is closest to your opinion about adolescents and alcohol?*). The parents were asked to mark one of three statements that represent their opinion: (1) Adolescents at my child's age are mature enough to handle alcohol in a responsible way; (2) I am not in favour of adolescents in the same age group as my child using alcohol, but I do not think adults can do anything about it and (3) To me it is obvious that adolescents under 18 years should not concern themselves with alcohol. The same question with a different response format demonstrated predictive validity in previous research such that restrictive attitudes were related to lower youth drinking [25].

#### Parents' behaviour regarding youth drinking

To measure parental behaviour regarding youth drinking the following question was used: "Has your child been offered alcohol at home?" The parent could mark one of five response alternatives: (1) No we don't drink alcohol in our family; (2) No; (3) Yes, he/she has been allowed to take a sip from a glass; (4) Yes, he/she has been served alcohol in their own glass; and (5) Yes, he/she has often been served alcohol in their own glass.

#### Parental education

Each year, parents were asked to report the highest educational level for themselves and for their partner. The responses were divided into three levels of education based on the seven response alternatives: "secondary school"; "university or university college, 2.5 years or less"; and "university or university college, 3 years or more". Mothers' and fathers' educational levels were examined separately.

#### Program participation rate

Parents were asked about the completion of the program activities in school years 8 and 9. It was not possible for the parents to state if they had completed one or two family dialogues in school year 7. The total numbers of activities that the parents could state that they had participated in were therefore 12 instead of all 13 that are included in the program. The 229 parents in the program group completed 3 activities on average. The number of activities completed allowed us to examine whether the rate of participation had an effect on program-related changes among parents and youth.

### Analysis

The design of the current study included data collection at three points in time (baseline, 15 months, and 27 months after the initiation of the program) allowing us to model change over time in the program and comparison group. The current data were analyzed using latent growth modelling (LGM) approach. LGM approach provides more flexibility in the analysis of change and complex representation of change process compared to traditional ANOVA approach for the analysis of repeated measure over time [36,37]. In addition, using Latent Growth Models (LGM) has been recommended for testing intervention effect by several pioneers of the area [37-40]. For designs with control or comparison groups, the suggested method is multiple-group LGM [36-38]. In single group LGM, the assumption is that the observations are obtained from a single population. Single group LGM approach would be suitable if the same developmental trajectory were expected to be observed across subgroups within the sample. However, in the current design, we expect a different developmental trend in parents' behaviours, youths drinking, and drunkenness in the program group due to the program effect compared to the non-participants. Multiple-group LGM model allows comparison of program and comparison groups for baseline differences, rate of change over time as well as differences in developmental pattern [37,40,41]. In addition, it is possible to examine different growth trajectories consistent with the hypothesized patterns of change across groups in multiple-group LGM models. In the current study, first, we fitted a multiple-group LGM model assuming linear growth for both program and comparison groups with no constraints on the intercept and slope factors. When the model does not fit the data, modifications can be made following hypothesized program effect. Specifically, the program was expected to postpone alcohol debut in the program group. Therefore, in the modified alcohol drinking model, we freely estimated the T2 alcohol drinking measure. Regarding drunkenness, we expected that there will be an accelerated increase in drunkenness in the comparison group. Therefore, we freely estimated, first T2 drunkenness, and later T3 drunkenness measures to identify the locale of the accelerated change. Second, after identifying the developmental pattern in both program and comparison groups, we added equality constraints to the intercepts across groups to test whether there were baseline differences in parents' behaviours, youth drinking, and drunkenness. Third, equality constraints were added to the slope factors to test whether the rates of change were different across the program and comparison groups. All these models were nested. Thus, the changes in the model fit were compared using chi-square difference test [42].

Parents' attitudes towards youth drinking were measured on two occasions in grades 7 and 9. Therefore, we used repeated measures ANOVA to test for the pre- and post-test differences in parental attitudes. In this model, parents attitudes were the within subject factor and groups (program vs. comparison) were the between subject factor.

Another purpose of the study was to examine whether the change pattern in the program group differ based on child gender, parent education, and number of parent-completed activities. For this purpose, centered (except gender) covariates were added to the growth model for the program group as time-invariant covariates [37,41].

The last purpose of the study was to test whether changes in parental attitudes were related to changes in alcohol use and drunkenness among youths. For this purpose, we fitted a cross-lagged model which includes measures of alcohol drinking, drunkenness, and parents' attitudes measured in Grade 7 (T1) and 9 (T2). In this model, the stability paths and cross-lagged paths were included. In addition, T2 measure of alcohol use and drunkenness were regressed on T2 parental attitudes, which now represents the change in parental attitude from T1 to T2 [43]. In this model, a significant cross-lagged path coefficient would suggest the effect of changes in parental attitudes on the changes in youth drinking and drunkenness.

We also estimated the program effect following the recommendations by Derzon and colleagues [44] for computing intervention effect size taking into account the differences between the intervention and control groups at baseline.

The study data was collected from 6 different schools. That is, the parents and students were clustered around schools. However, the number of clusters was not sufficient for using multilevel modelling techniques. Therefore, we included dummy school codes in all models to control for the possible variations due to clustering. All models were fitted with Full Information Maximum Likelihood estimator using MPlus 4.21 [45]. In all analyses, the proportion of available data ranged between 72% to 100%, and the average was 85% across the analyses.

## Results

### Baseline Differences

The parents and teenagers who participated in the program were compared to the non-participants to examine baseline group differences on major study variables. The only difference was with regard to maternal education, in that mothers who participated in the program had significantly higher education than the non-participating mothers (see Table 2). There was no other significant group difference.

**Table 2:** Means and standard deviations of baseline measures, and group differences at baseline.

	Intervention Group		Comparison Group		*t*	*p*
	
	Mean	STD	Mean	STD		
Mother's education	2.00	.96	1.82	1.03	2.12	.04
Father's education	1.70	1.02	1.56	1.00	-1.77	.08
Parents' attitudes	2.83	.42	2.81	.41	-.53	.60
Parents' behaviors	2.30	.55	2.33	.58	.50	.62
Youth drinking	.15	.36	.19	.40	1.24	.22
Youth drunkenness	.04	.48	.08	.59	1.69	.09

STD: Standard deviation

### Program Effect on Parents' Behaviours and Attitudes

The baseline multiple-group LGM model with no constraints on intercept and slope factors, and linear growth assumption for parents' behaviour fitted the data with a non-significant model fit chi-square, *χ*^2^(2) = 3.802, p = .15. However, the mean of slope factors in both groups were also non-significant suggesting that there was no significant change in parents behaviours over time. To test whether there were differences at the baseline, equality constraints to intercepts were added. There was no significant change in model fit due to equality constraints on intercept, suggesting that the program and comparison group parents did not differ in serving alcohol to their youths (*χ*^2^(3) = 5.630, p = .13). Even though the slope factors had non-significant mean values, they had opposite signs (-.004 for the program and .004 for the comparison groups). Therefore, equality constraints were added to the slope means to test whether there were significant differences. The model with equality constraints on slope factor fitted the data well suggesting that the trend of change in parents' behaviours were not different (*χ*^2^(3) = 6.024, p = .11). In sum, there was no significant effect of program on parents' behaviours.

**Figure 1:**
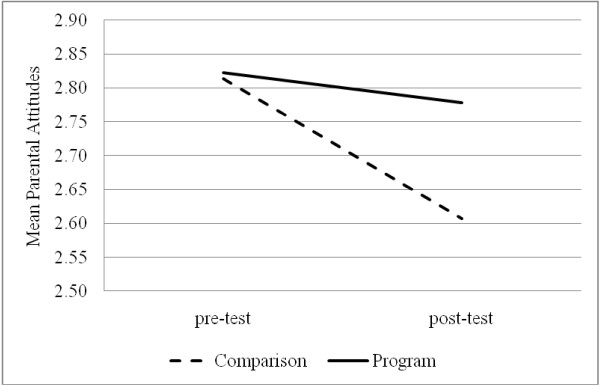
**Pre-post test change in parental attitudes following the program**.

The changes in parents' attitudes regarding youth drinking were examined with a mixed design ANOVA model with the groups (program vs. comparison) as the between group and pre- and post-test measures of parental attitudes as the within-group factor. The results suggested that there was a change in parental attitudes (*F*(1, 323) = 19.64, *p *< .001) and that the change was affected by the program (*F*(1, 323) = 8.20, *p *< .01). Specifically, parents participating in the program maintained their restrictive attitudes, while the parents in the comparison group adopted more lenient attitudes towards youth drinking over time (*d *= .32) (see Figure 1).

### Program Effect on Youth Alcohol Use

The program effect on youth alcohol consumption was examined in two domains: drinking alcohol and drunkenness. Alcohol drinking becomes an increasingly common behaviour among young people as they get older, and most of those who drink alcohol do not develop problem behaviours as a result of alcohol consumption. Drunkenness, however, represents a problem behaviour which is of great concern for preventive efforts. Therefore, we examined the effect of the program on drinking and on drunkenness separately.

**Figure 2:**
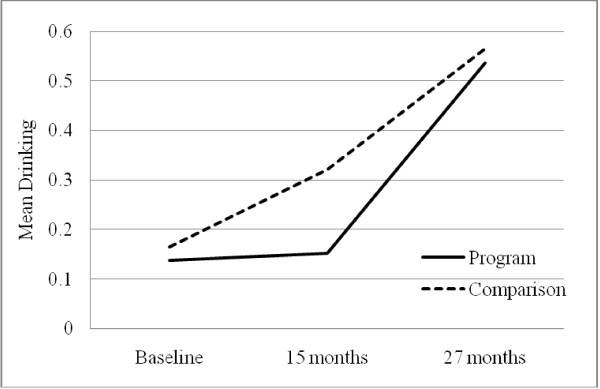
**Change in youth drinking in the program and comparison groups**.

The multiple group LGM model assuming linear change in drinking for both groups did not fit the data, *χ*^2^(2) = 10.80, p = .005. Because the program goal was to postpone drinking debut, we freely estimated the T2 slope indicator for the program groups. No change was made in the comparison group. This modification significantly improved the model fit (Δ*χ*^2^(1) = 5.45, p = .02) with good fit indices (CFI = .98, RMSEA = .09, SRMR = .03). The results suggested that the growth pattern for the comparison group was linear whereas the growth for the program group was quadratic (see Figure 2). Drinking in the comparison group steadily increased from baseline to 27-months following the initiation of the program. However, drinking in the program group remained relatively stable and then increased towards T3, suggesting that the intervention postponed alcohol debut age in the program group. The test for the baseline differences in drinking by adding equality constraints suggested that there were no group differences when the program was initiated. The rates of change from 15 months to 27 months were not significantly different for the program and comparison groups as well (*d *= .08).

**Figure 3:**
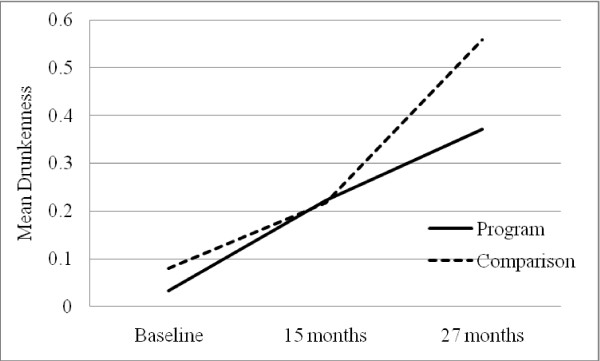
**Change in youth drunkenness in the program and comparison groups**.

The linear LGM model for drunkenness did not fit the data as well, *χ*^2^(2) = 27.81, p < .001. We expected that the comparison group would increase drunkenness at a faster rate than the program group. Thus, we freely estimated the T3 slope indicator for the comparison group. This new model revealed significantly improved model fit (Δ*χ*^2^(1) = 21.5, p < .001). The results suggested that the changes in drunkenness among comparison group youths followed a quadratic pattern with accelerated increase from Grade 8 to Grade 9. On the other hand, there was a linear increase in drunkenness in the program group (see Figure 3). The comparison group had a slightly higher, but non-significant drunkenness rate at the baseline compared to the program group. However, drunkenness rate by T3 was lower for the program group than the comparison group adolescents (*d *= .13).

### Effect of Youth Gender, Parent Education, and Number of Parent-Completed Activities

The effects of youth gender, parental education (i.e., mother's and father's level of education), and number of parent-completed activities were examined on both process (i.e., serving alcohol and attitudes towards youth drinking) and outcome variables (i.e., drinking and drunkenness). All three variables were included in the LGM models as time invariant covariates one at a time. The models were tested only on the program group. The covariates significantly predicted neither intercept nor slope factor of the LGM models, suggesting that the initial level and change patterns were not different for boys and girls, and at varying levels parent education and participation rate. The same variables were used as covariates of parental attitudes in the mixed design repeated measures ANOVA model as well. None of them significantly interacted with the change in parental attitudes over the course of the intervention. Overall, the findings suggest that the program effect was not sensitive to gender of the youth, parental education, and the number of parent-completed activities.

### The Effects of Parents' Behaviours and Attitudes on Youth Drinking and Drunkenness

The program theory of Strong and Clear was that the changes in parents' behaviour and attitudes would lead to reduced drinking and drunkenness among their children. That is, the effect of the program on the outcomes would be mediated by the process variables. Nevertheless, the program had no effect on parents' behaviour, violating the assumption of mediation effect [46]. Regarding parental attitudes, the pre-post measurement design did not allow using the LGM framework to test the mediation effect of parental attitudes within LGM framework [40]. Instead, two separate cross-lagged regression models were fitted to test whether changes in the participating parents' attitudes towards youth drinking predicted changes in drinking and drunkenness among youths. In these models, the T2 measures of alcohol use variables were regressed on both their T1 measures and the T2 parental attitude. Parental attitude and alcohol use variables at T2 represent the residual change from T1 to T2 in the respective variables. That is, a significant path coefficient from T2 parental attitudes to alcohol drinking or drunkenness would suggest a significant effect of changes in parental attitudes on youth outcomes.

The results suggested that changes in parental attitudes negatively predicted drinking (*β *= - .21, *z *= -2.61, *p *< .01) and drunkenness (*β *= -.22, *z *= -2.57, *p *< .01) at T2 for the program group. Nevertheless, these paths were not significant for the comparison groups. Overall, the findings suggest that the restrictive attitudes of parents predicted lower levels of drinking and drunkenness among youths.

## Discussion

Strong and Clear aims to maintain parents' restrictive attitudes towards youth drinking, and in turn, prevent alcohol drinking among adolescents. Because drinking is common among Swedish adolescents, it is not realistic to expect that the program will stop teen drinking. An effective program, however, may postpone the onset of drinking and reduce drunkenness rates. The main findings of the study suggest that adolescents whose parents participated in the Strong and Clear program made their alcohol debut about one year later than other adolescents and had a lower rate of drunkenness by the end of the intervention. These results correspond to some degree with the results of the previous evaluation of the program in Norway. For example, Bolstad and colleagues [31] also found that adolescents whose parents participated in the program made their alcohol debut about one year later than the adolescents of the non-participating parents. Contrary to the present study, they found that participating parents were less likely to offer alcohol to their child at home.

One of the important findings of the current study is that the participating adolescents' delayed alcohol debut and reduced drunkenness were related to parents' restrictive attitudes towards youth drinking. This finding is consistent with previous studies suggesting a significant association between parental attitudes and adolescent alcohol use [13-18]. There has been also successful preventive interventions that include components to strengthen parents' restrictive attitudes toward youth and alcohol [15,25]. Strong and Clear also included a component where parents were encouraged to make an agreement with each other about urgent questions in the teenage period, like values about alcohol shared in common with other parents. Agreements between parents about their position concerning youth drinking have been used in other studies [25,47]. A limitation of the present study was that parents' attitudes were only measured at two time-points, before and after the program. Therefore the mediating effect of changes in parental attitudes could not tested through LGM approach which could allows analysis of multiple change process and mediating effects simultaneously [40]. However, it should be noted that cross-lagged models are also suitable to test predictive role of change in parents' attitudes between two time points on changes in youth drinking [43].

An alternative explanation for the program effect could be related to the emphasis on the family-strengthening activities such as the family dialogues. Overviews have shown that effective interventions often aim to improve family interactions and bonding [15,24]. The activities that required involvement of both parents and adolescents could also have played a role on the effect of the program. Koning and colleagues [47] tested the differential outcomes of three intervention conditions: parents only, adolescents only, and parents-adolescents combined. Neither the parental intervention nor the adolescents only condition was effective in delaying the onset of weekly alcohol use or reducing the frequency of drinking. However, the intervention was effective when both of the components were combined. In the evaluation of Strong and Clear it was not possible to analyze the impact of each component separately. Even though the research team was determined for implementation of the program with high fidelity, the family meetings and the friend meetings were carried out by only a few families. For this reason, the present evaluation could not include an analysis of the effects of each component in the program. Understanding which components of the program contributed to the change would have been of great value for improving the current program, as well as for other prevention programs under development.

The effects of a program may change based on some program-related factors or background characteristics of the participants. Therefore, the effects of gender, parental education, and program participation rates were examined in the present study. The results did not suggest any difference in changes in the program group due to adolescent gender. Kumpfer and colleagues [24], in a review about prevention programs for substance use, emphasized the importance of investigating the effect of programs across gender. They found that girls are more influenced by family protective factors than boys. Strong and Clear did not have a stronger effect for girls, suggesting that both boys and girls showed a changes pattern over time in alcohol drinking. There was also no relation between parental education and the effect of program on participating parents and youths.

Strong and Clear is a relatively comprehensive program, comprising 13 activities over three years. Nevertheless, parents in the program group participated relatively few number of program related activities during the three years of secondary school. Interestingly, we could not find any significant association between participation rate and program effect on the changes in both parents and youths behaviours. It is possible that maintaining active participation in all or most activities was not crucial for the program effect. Instead, a specific type of activity where parents or youths involve, or maintaining restrictive attitudes could be the important components of the program that drives change. Indeed, Koning and colleagues [47], as previously mentioned, have shown that programs involving both parents and youths were more effective than parent-only or adolescent-only programs. In addition, Koutakis and his colleagues [25], in their randomized-controlled-trial, have shown that helping parents maintain restrictive attitudes towards youth drinking was highly effective in reducing problem drinking among Swedish youths. It is essential to further examine which components of Strong and Clear, and how much involvement in its activities are important for observing program effect.

One advantage of this program is that a non-governmental organization implements the program. The Swedish voluntary sector, especially the temperance movement, has a long tradition of alcohol prevention [48]. The important role of the voluntary sector is emphasized in the Swedish action plan on alcohol and illicit drugs as well as in the government bill for public health. It is also pointed out that the NGOs have the capacity to complement and strengthen the work of the public sector [49,50]. While the municipalities have limited resources and difficulties in engaging staff after work hours, the NGOs often have personnel resources that work on a voluntary basis in their leisure-time. Many parental support programs involve schools to some extent. The Swedish National Agency for Education has emphasized that it is not a primary task of the schools to arrange these kinds of interventions [51]. Therefore, the NGOs could be an important provider of parental programs, as they are independent of the school system. Schools might be an arena for prevention programs, but NGOs could just as well implement programs in other contexts like sports organizations or youth recreation centres.

### Strengths and limitations

An important strength of the present study is that it included repeated measurements of both adolescents and parents during the three years of secondary school. There is a need for longitudinal studies to track both the initiation and process of alcohol consumption among youth [52]. At least three time-points are necessary to investigate individual differences in change over time [40].

An additional strength is that the analysis includes both alcohol drinking and drunkenness. Spoth and colleagues [52], in an overview of preventive interventions addressing underage drinking, assert that prevention trials should use specific measures of outcomes. They state that many trials are unspecific and assess substance use in general. Preventive interventions may impact some specific outcomes but not others. Therefore, a variety of well-defined measures are essential to conduct rigorous evaluations of prevention programs.

The National Board of Health and Welfare have stated that one of the goals of the funding to NGOs was to develop knowledge about alcohol and drug prevention methods and to produce evidence for the effects of different interventions [53]. The present study has contributed to strengthening the evidence-based practice by evaluating an intervention under typical, rather than optimal conditions, which is important for the external validity [29]. In Sweden, NGOs are important actors within alcohol and drug prevention, and undertake comprehensive efforts [54]. Nevertheless, only a limited number of NGO-developed and -implemented interventions have been evaluated thoroughly. Thus, there is an urgent need to develop an intervention research agenda focusing on prevention programs develop and implemented by NGOs [27,28].

The present study is not a randomized controlled trial, which has been considered the gold standard in evaluations of interventions [55,56]. It was not possible for IOGT-NTO to implement the program in a defined geographical area due to the grant requirements of the Swedish National Board of Health and Welfare. The evaluation of the program had to be concentrated to areas where the organization had enough personnel and organizational resources to carry out the program on a sufficiently large scale. IOGT-NTO was initially guaranteed funds for two years, after which they needed to apply for supplemental funding for each additional year. In addition, schools could only be included in the evaluation on a voluntary basis and only six schools declared an interest in participating. The uncertainty regarding continued funding, and the limited number of schools interested in participating in the evaluation limited our ability to conduct a randomized controlled trial.

Parents in the analytical sample might differ from Swedish parents in general but it is also possible that they differ from parents in the three municipalities where the families lived. For example, the parents in the study had relatively high education level and were primarily mothers. However, this trend is highly similar to previous studies. Previous studies examining the attrition in longitudinal studies have shown that people staying in a longitudinal study often have higher level of education than those lost through attrition [e.g. [57,58]]. In addition, Swedish women tend to respond postal questionnaires at a higher rate than Swedish men Lindén-Boström and Persson [2010, unpublished data]. Mothers are also more likely to take part in parent-focused programs [e.g. [59-61]].

It is also possible that the adolescents within the analytical sample differed from Swedish adolescents in general. The year 9 questionnaire was conducted about two months after a Swedish national survey among year 9 students [62]. When the analytical sample and the national survey were compared, it emerged that the girls in the analytical sample consumed somewhat less alcohol than Swedish girls in general, while the boys in the analytical sample had been drunk to a somewhat greater extent than the national average.

Despite some methodological limitations, the present study has achieved the goal of the funding from the NBHW to develop knowledge about alcohol and drug prevention methods and to produce evidence for the effects of different interventions [53].

### Future research

Despite the promising results of the present evaluation and a previous evaluation [31], more research is needed to further evaluate the program. First, it would be highly useful to conduct randomized-controlled-trials to further examine the efficacy of the program. Second, there a is an urgent need to test the contribution of each components to program activities. Strong and Clear is a relatively comprehensive program and the results are not too overwhelming. The present study also showed that it was difficult to engage parents in all activities in the program, especially the family meetings and the friend meetings. Examining the importance of the different components of the program and the mediating role of parental attitudes and behaviours in rigorous randomized controlled trails would be useful in modifying or further improving the program. Such studies may also contribute to developing a more cost effective version of the program. Third, it would also be interesting to follow the participants of the program to identify longer term effects of the program beyond adolescence. Alcohol consumption, especially among men, reaches its peak during young adulthood. Young men have their highest consumption in their early 20 s, and drink more than twice as much as women in the same age group. From about 25 years of age, men's consumption falls while that of women stabilizes at a lower level [63]. Further research should also consider the gender aspect of the participating parents. Even though Kumpfer and colleagues [24] have asserted that a program might have different effects depending on the gender of the adolescents, no attention has been paid to the effect of gender of participating parent. The impact of parental gender may be of particular importance in countries such as Sweden, where gender gap in parenting is lower compared to many of its counterparts.

## Conclusions

The results of the study suggested that Strong and Clear contributed to maintaining parents' restrictive attitudes towards underage drinking during secondary school, postponing alcohol debut among the adolescents, and significantly reducing their drunkenness.

## Competing interests

The authors declare that they have no competing interests.

## Authors' contributions

CP was the main author of the manuscript and was involved in all aspects of the study. MÖ was involved in the analysis and the writing of the manuscript. CE was involved in the design of the study and he provided scientific oversights and feedback throughout the development of the study and the manuscript. All authors read and approved the final manuscript.

## Pre-publication history

The pre-publication history for this paper can be accessed here:

http://www.biomedcentral.com/1471-2458/11/251/prepub
